# How Do People Process Different Representations of Statistical Information? Insights into Cognitive Effort, Representational Inconsistencies, and Individual Differences

**DOI:** 10.1177/0272989X231202505

**Published:** 2023-10-16

**Authors:** Kevin E. Tiede, Wolfgang Gaissmaier

**Affiliations:** Center for Adaptive Rationality, Max Planck Institute for Human Development, Germany; Department of Psychology, University of Konstanz, Germany; Graduate School of Decision Sciences, University of Konstanz, Germany; Department of Psychology, University of Konstanz, Germany; Centre for the Advanced Study of Collective Behaviour, University of Konstanz, Germany

**Keywords:** cognitive effort, decision aids, graph literacy, icon arrays, numeracy, risk communication

## Abstract

**Background:**

Graphical representation formats (e.g., icon arrays) have been shown to lead to better understanding of the benefits and risks of treatments compared to numbers. We investigate the cognitive processes underlying the effects of format on understanding: how much cognitive effort is required to process numerical and graphical representations, how people process inconsistent representations, and how numeracy and graph literacy affect information processing.

**Methods:**

In a preregistered between-participants experiment, 665 participants answered questions about the relative frequencies of benefits and side effects of 6 medications. First, we manipulated whether the medical information was represented numerically, graphically (as icon arrays), or inconsistently (numerically for 3 medications and graphically for the other 3). Second, to examine cognitive effort, we manipulated whether there was time pressure or not. In an additional intervention condition, participants translated graphical information into numerical information before answering questions. We also assessed numeracy and graph literacy.

**Results:**

Processing icon arrays was more strongly affected by time pressure than processing numbers, suggesting that graphical formats required more cognitive effort. Understanding was lower when information was represented inconsistently (v. consistently) but not if there was a preceding intervention. Decisions based on inconsistent representations were biased toward graphically represented options. People with higher numeracy processed quantitative information more efficiently than people with lower numeracy did. Graph literacy was not related to processing efficiency.

**Limitations:**

Our study was conducted with a nonpatient sample, and the medical information was hypothetical.

**Conclusions:**

Although graphical (v. numerical) formats have previously been found to lead to better understanding, they may require more cognitive effort. Therefore, the goal of risk communication may play an important role when choosing how to communicate medical information.

**Highlights:**

- This article investigates the cognitive processes underlying the effects of representation format on the understanding of statistical information and individual differences therein.
- Processing icon arrays required more cognitive effort than processing numbers did.
- When information was represented inconsistently (i.e., partly numerically and partly graphically), understanding was lower than with consistent representation, and decisions were biased toward the graphically represented options.
- People with higher numeracy processed quantitative information more efficiently than people with lower numeracy did.

Patients have to understand the benefits and risks associated with medical treatments in order to make informed medical decisions. To help patients with this challenging task, decision aids have been developed^1–4^; these often present the frequencies of benefits and side effects graphically, for instance, as icon arrays or bar plots. The use of graphical representation formats is recommended by the International Patient Decision Aid Standards (IPDAS)^5^ because a multitude of studies has shown that graphical representation formats lead to better understanding of medical information compared with numerical representation formats (for reviews, see Garcia-Retamero and Trevena et al.^5^, Cokely,^6^ and Spiegelhalter^7^), but less is known about *how* different representations affect understanding. Understanding how people process information depending on its representation format can help to design decision aids. Therefore, in this article, we investigate the cognitive processes underlying the effects of numerical and graphical representations on understanding. Specifically, we examine how much cognitive effort is required to process icon arrays and numbers, how people process inconsistently represented information (i.e., information represented partly numerically and partly graphically), and how individual differences in numerical and graphical abilities relate to differences in the processing of numerical and graphical information. We do this by testing a series of confirmatory hypotheses (Hs) and exploratory research questions (RQs).

## Numerical versus Graphical Representations

People have been shown to understand medical information better when it is presented graphically compared to numerically.^5–7^ However, less attention has been paid to how people cognitively process different representations—in particular, the cognitive effort required to process graphical and numerical information and whether one is easier to process than the other. One way to capture cognitive effort is to measure response times,^8-10^ with longer response times corresponding to more cognitive effort.^11–14^ Studies have shown that people take more time to respond to questions when they work with graphical formats than with numbers^15,16^ (but see Brewer et al.^17^). However, the correlational design of these studies could not test why people deliberated longer with graphs than with numbers. For instance, people could merely *take* more time when provided with icon arrays because they are more engaged or they could *need* more time because icon arrays are harder to process. Our study aims to disentangle these accounts by experimentally examining the understanding of information when time is limited. When there is no time pressure, longer response times with icon arrays could indicate increased engagement, increased cognitive effort, or both. If longer response times can be at least partly attributed to increased cognitive effort due to less efficient information processing, limiting the time to read the information and answer questions should have a stronger detrimental effect on understanding for icon arrays than for numbers. To investigate how much cognitive effort icon arrays and numbers require, we implement conditions with and without time pressure and first examine whether understanding (RQ1) and response times (RQ2) differ when information is represented numerically versus graphically when there is no time limit. We then test our assumption that when there is time pressure, understanding is worse than when there is no time pressure (H1) and examine whether time pressure decreases understanding more strongly with graphically (v. numerically) represented information (RQ3).

## Consistent versus Inconsistent Representations

Another approach to investigating how people process graphical and numerical representations is to examine how they process inconsistently represented information—that is, when information is represented partly numerically and partly graphically. This approach can reveal how people internally represent numerically and graphically represented information when they need to integrate information that is in different formats. Theories of graph comprehension (e.g., Pinker^18^) assume that numbers and graphs are internally represented differently, and thus, an additional translation step would be necessary to compare them. We test how people internally represent inconsistently formatted information: if people internally represent the information similarly (e.g., by counting the number of icons in an icon array and mentally representing all information as numbers^19,20^), understanding should be comparable to when information is formatted consistently (e.g., purely numerically). However, if people’s internal representations of information differ even when comparing information, understanding should be worse when options are represented inconsistently than when they are represented inconsistently (H2). We also hypothesize that integrating inconsistent representations is a cognitively demanding task that requires more effort than merely comprehending each piece of information, and therefore, response times are longer (H3) and time pressure decreases understanding more strongly when information is represented inconsistently than when it is represented consistently (H4).

Furthermore, we test whether people deal with inconsistent representation by translating the information from one representation into another before comparing options. To that end, the study also includes an intervention condition in which people are asked to read graphically represented information and enter it numerically on the survey page before answering questions about the information. If people generally process inconsistent information by performing this translation, there should be no difference in understanding between the conditions with and without this intervention. However, we hypothesize that people do not perform this translation despite being capable of doing so, and thus, understanding is better with the intervention than without (H5).

Moreover, we investigate whether information is evaluated differently depending on its format. People have been shown to prefer graphical over numerical representation formats^21,22^ and to allocate more attention to graphical than numerical formats.^16^ More attention to an option, in turn, increases the likelihood of choosing it.^23–25^ This could lead to a more favorable evaluation of a graphically represented option, so that people choose graphically represented options more often, independent of the actual information. Therefore, we hypothesize that decisions are biased toward the graphically represented options in the inconsistent conditions (H6).

## Numeracy and Graph Literacy

People may also systematically differ in how they process numerically and graphically represented information due to their cognitive abilities. Numeracy,^26,27^ the ability to understand and use numerical and probabilistic information, is not only positively related to the comprehension of risk information^28,29^ but also affects how people process such information. When provided with numerical information, people with higher numeracy deliberate longer^30,31^ and look longer and more frequently at the information compared with people with lower numeracy.^32,33^ On the other hand, one study found that people who rated themselves as having higher numeracy spent less time looking at risk information, suggesting that they process information more efficiently than people who rated themselves as having lower numeracy.^34^ In the present study, we investigate whether numeracy is positively related to understanding (RQ4a) and response times (RQ5a) when there is no time limit, as well as whether time pressure decreases understanding less for people with higher numeracy than those with lower numeracy (RQ6a).

Numeracy has also been found to be related to the processing of icon arrays. More numerate people tend to count icons, whereas less numerate people tend to process the array holistically.^19,20^ Because counted icons, rather than representations of highlighted versus nonhighlighted areas, are easier to compare with numbers, people with higher numeracy should benefit from the intervention less than people with lower numeracy, which consists of translating graphical into numerical information. We therefore test whether the effect of the intervention on understanding is moderated by numeracy (RQ7).

Finally, people with higher graph literacy,^35^ the ability to understand graphically presented information, have been shown to better comprehend icon arrays and to be better at identifying task-relevant information in graphs compared with people with lower graph literacy.^35,36^ Analogously to numeracy, we investigate whether graph literacy is positively related to understanding (RQ4b) and response times (RQ5b) when there is no time limit, as well as whether time pressure decreases understanding less for people with higher graph literacy than those with lower graph literacy (RQ6b).

## The Present Study

The present research examines how people process medical information when it is represented numerically, graphically, or inconsistently (i.e., some options numerically, others graphically) and how people differ in the processing of this information. We provided participants with the relative frequencies of benefits and side effects of 6 treatments represented in different ways (and, in the intervention condition, asked participants to translate graphical information into numerical information). To measure understanding, we assessed decision accuracy by asking participants to choose their preferred treatment—2 of the 6 treatments were superior (i.e., Pareto optimal)—and assessed knowledge by asking participants questions about the medications. We also manipulated whether there was a time limit to answer the questions. Finally, we assessed numeracy and graph literacy.

We preregistered the study, RQ1 and RQ6, and all stated confirmatory Hs as well as their analyses at OSF (https://osf.io/h37ej). Table 1 lists all RQs and Hs tested in this study. All preregistered RQs and Hs focused on response times and decisions as outcome variables, yet we studied all RQs and Hs regarding decisions with knowledge as an outcome variable as well.

**Table 1:** Summary of Research Questions and Tested Hypotheses with Results^a^

No.	Hypothesis (H)/Research Question (RQ)	Result: Decision	Result: Knowledge	Preregistered
Effect of time pressure				
H1	When there is time pressure, understanding is worse than when there is no time pressure.	**✓**	**✓**	Yes
Numerical versus graphical representations				
RQ1	Does understanding differ when information is represented numerically v. graphically?	×	**✓**	Yes
RQ2	Do response times differ when information is represented numerically v. graphically?	**(✓)**	**✓**	No
RQ3	Does time pressure decrease understanding more strongly when information is represented graphically (v. numerically)?	×	**✓**	No
Consistent versus inconsistent representations				
H2	When medication options are represented inconsistently, understanding is worse than when they are represented consistently (i.e., fully numerically or graphically).	**✓**	**(✓)**	Yes
H3	When there is no time limit, response times are longer with inconsistently (v. consistently) represented information.	**✓**	**(✓)**	Yes
H4	When medication options are represented inconsistently, time pressure (v. no time pressure) decreases understanding more strongly than with consistent information.	×	**(✓)**	Yes
H5	With inconsistent representation, understanding is better with an intervention that prompts people to translate graphical into numerical information than without an intervention.	**✓**	×	Yes
H6	With inconsistent representation, decisions are biased toward the graphically (v. numerically) represented options.	**✓**	n/a	Yes
Numeracy and graph literacy				
RQ4	Is understanding positively related to (a) numeracy and (b) graph literacy?	a: **(✓)** b: **(✓)**	a: **(✓)** b: **(✓)**	No
RQ5	Are response times positively related to (a) numeracy and (b) graph literacy?	a: × b: ×	a: **(✓)** b: ×	No
RQ6	Does time pressure decrease understanding less strongly for people with higher (v. lower) (a) numeracy and/or (b) graph literacy?	a: × b: ×	a: **✓** b: ×	Yes
RQ7	Does numeracy moderate the effect of intervention on decisions so that the intervention improves accuracy less strongly for people with higher (v. lower) numeracy?	×	×	No

a **✓** = Hypothesis confirmed/research question affirmed. × = No support for hypothesis/research question. (**✓**) = Partial support for hypothesis/research question. n/a = Not applicable.

## Methods

The study was approved by the ethics committee of the University of Konstanz, Germany.

### Design

All participants were provided with information on 6 hypothetical medications to treat multiple sclerosis (MS; see Table 2; for screenshots of instructions, see Supplementary Figure S1) and were asked questions about them while the information was visible. The information showed how many of 100 people experienced benefits and side effects. The relative frequencies were constructed such that 1 medication was dominant in each of 2 triplets of medications (i.e., Pareto optimal: more frequent benefits with equally or less frequent side effects, or less frequent side effects with equally or more frequent benefits), resulting in 2 dominant options across all 6 medications. A medication of 1 triplet did not dominate the medications of the other triplet. This approach ensured that there were objectively superior medications without rendering the decision obvious and that 1 of the 2 superior medications was always dominant, even when considering other criteria of superiority (e.g., greatest benefit–risk ratio).

**Table 2:** Medical Data Used in the Study^a^

	Benefits	Side Effects
**Medication 1**	**67 out of 100**	**31 out of 100**
Medication 2	67 out of 100	37 out of 100
Medication 3	63 out of 100	34 out of 100
**Medication 4**	**46 out of 100**	**21 out of 100**
Medication 5	41 out of 100	21 out of 100
Medication 6	43 out of 100	25 out of 100

a The 2 dominant options are printed in boldface. Medication 1 dominates medications 2 and 3 because it has more frequent benefits with at most equally frequent side effects or less frequent side effects with at least equally frequent benefits (i.e., it is Pareto optimal). For the same reason, medication 4 dominates medications 5 and 6. Allocation of each medication to each of the 6 positions on the page was randomized across participants (see also Figure 1).

While the medical information was the same for all participants, the way the information was presented differed between conditions. In the numerical conditions, relative frequencies were presented as numbers (e.g., “67 out of 100”; see upper row of Figure 1 and Supplementary Figure S2). In the graphical conditions, relative frequencies were presented as icon arrays (see lower row of Figure 1 and Supplementary Figure S3). In the inconsistent conditions, information on 3 medications was presented as numbers and information on the other 3 medications was presented as icon arrays (see Figure 1; placement of numbers in the upper or lower row was randomized across participants).

**Figure 1:**
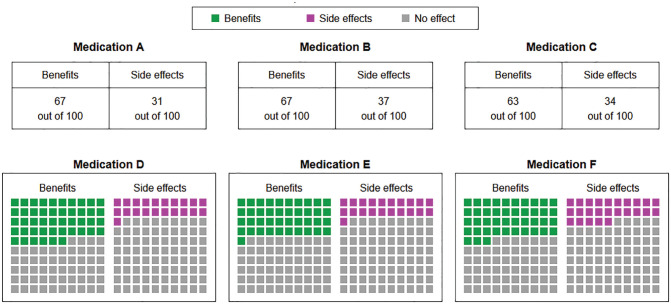
Medical information as presented in the inconsistent conditions. In the time-pressure conditions, a countdown was visible in the lower-right corner. The placement of the medications was randomized across participants; here, Medication A dominates B and C, and Medication D dominates E and F.

We also manipulated between participants whether there was time pressure or not. In the time-pressure conditions, participants had a limited amount of time to answer each question. Because a pilot study showed that response times differed systematically between question types, the time limit ranged from 15 to 30 s (see Table 3; the pilot study is described in the Supplementary Material). These time limits reflected the time in which about half of participants answered the questions without a time limit in the pilot study. The allowed time for each question was announced before each question page and the remaining time was presented on the question page next to the question (for screenshots, see Supplementary Material). When the time was up, the study automatically proceeded to the next page. In the no-time-pressure conditions, there was no time limit.

**Table 3:** Items Used in This Study and Respective Time Limits in the Time-Pressure Conditions

Question Type	Question	Time Limit
Decision	Considering both the benefits and side effects, which of the medications would you prefer?	30 s
Gist knowledge	With which medication did people experience benefits most often?	15 s
Gist knowledge	With which medication did people experience side effects most often?	15 s
Verbatim knowledge (identification)	If 100 people take medication E, how many of them will experience benefits?	15 s
Verbatim knowledge (identification)	If 100 people take medication C, how many of them will experience side effects?	15 s
Verbatim knowledge (computation of differences)	If 100 people take medication [A/B],^a^ how many more of them will experience benefits compared to Medication [A/B]*?	25 s
Verbatim knowledge (computation of differences)	If 100 people take medication [D/F],^a^ how many more of them will experience side effects compared to medication [D/F]^a^?	25 s
Verbatim knowledge (computation of differences)	If 100 people take medication [C/D],^a^ how many more of them will experience benefits compared with medication [C/D]^a^?	25 s
Verbatim knowledge (computation of differences)	If 100 people take medication [A/E],^a^ how many more of them will experience side effects compared to medication [A/E]^a^?	25 s

a In the questions testing verbatim knowledge (computing-differences), the medication with the higher frequency of benefits or side effects was stated first and the other medication second, so that the correct answer was always positive. Medication labels (i.e., A to F) refer to the location of the medication (e.g., medication A is the upper-left medication).

**Figure 2:**
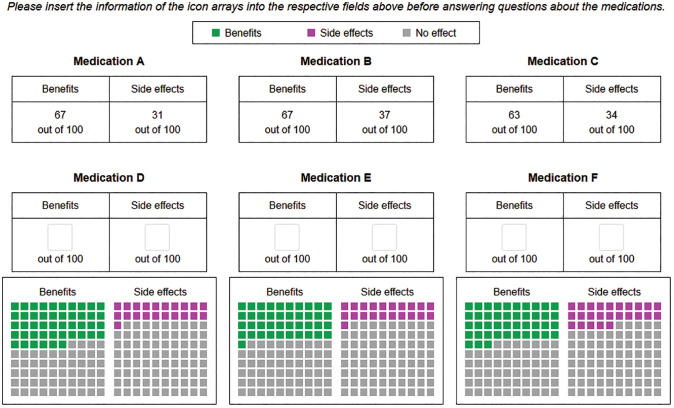
Screenshot of the intervention condition. Before answering questions, participants were asked to read the graphical information and to fill in the frequencies into the respective fields. After that, participants were provided with the original and the translated information.

**Figure 3:**
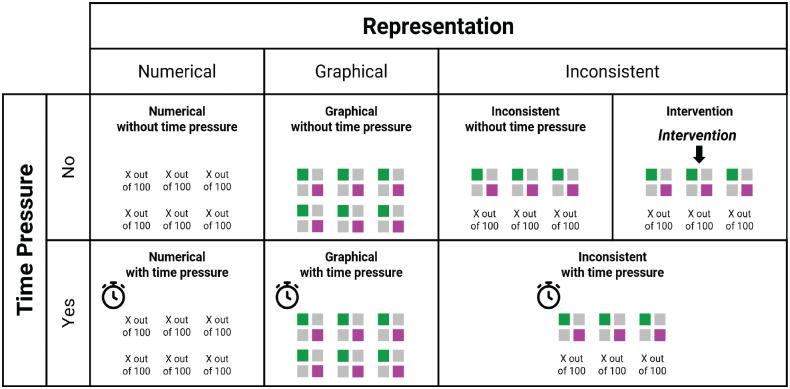
Overview of conditions of the study.

In addition to these 2 experimental factors, there was an intervention condition in which participants received the same information as in the inconsistent conditions and had no time limit, but before answering the questions, they were asked to read the graphically represented information and enter the respective numeric frequencies into text boxes below or above the icon arrays (see Figure 2). Subsequently, participants were provided with both the original information and their numerical entries when answering the same questions as in the other conditions (see Supplementary Figure S4). A schematic illustration of the conditions in this study is presented in Figure 3.

The 6 medications from Table 2 were randomly allocated for each participant to avoid any potential effects of placement on choice. This also ensured that in the inconsistent conditions, it was equally likely for each of the 2 superior medications to be represented numerically or graphically. Exploratory analysis revealed that there was no effect of whether the 2 superior options were represented in the same versus different format.

In sum, we implemented a 3 (format: numerical v. graphical v. inconsistent) × 2 (time pressure: yes v. no) between-subjects design with an additional intervention condition. The experimental factors were independent variables, whereas decision accuracy, knowledge, and response times were dependent variables.

### Procedure

The study was held online; participants used a computer and provided all answers with their mouse cursor or keyboard. After providing informed consent, participants filled out questionnaires on subjective numeracy^37^ (i.e., numeric confidence and numeric preferences) and subjective graph literacy^38^ (i.e., graph-related confidence), which were not included in the main analysis. Participants were randomly assigned to 1 of the 7 conditions. Once they had seen the instructions, they were presented with information on 6 medications and asked which they would choose if diagnosed with MS. Next, participants were asked 8 knowledge questions in randomized order, with the medical information always visible. Subsequently, participants completed measures on numeracy and graph literacy and provided demographic information. Finally, they were asked about whether they had taken their participation seriously and were debriefed.

### Measures

A list of all decision and knowledge items can be found in Table 3.

#### Decision

After being instructed to imagine being diagnosed with MS, participants were asked to choose the medication they preferred. A decision was counted as correct if 1 of the 2 dominant medication options was chosen and, in the time-pressure condition, if the participant answered within the time limit.

#### Knowledge

We assessed knowledge using 8 items that asked about gist knowledge (i.e., understanding of the essential information^39^) and verbatim knowledge (i.e., precise quantitative knowledge), based on measures from previous research.^40–44^ We measured gist knowledge by asking for ordinal comparisons of the risks and benefits of the medications. Two items asked participants to identify the medication with the least frequent benefits or the most frequent side effects by choosing 1 of the 6 medications in a multiple-choice format. We measured verbatim knowledge using 2 items asking participants to identify information about benefits and side effects and 4 items asking participants to compute the differences in benefits or side effects between 2 medications. Two of these 4 items asked for comparisons between medications within the same row (i.e., the same format in the inconsistent conditions), and the other 2 items asked about medications in different rows (i.e., different formats in the inconsistent conditions). Participants answered verbatim knowledge questions by typing a number into a text box. All verbatim knowledge items referred to medications at the same location across participants (e.g., the upper-right “medication C”), but the allocation of medications was randomized, so correct answers differed between participants. The gist and verbatim knowledge scores represent the proportion of answers across the 2 and 6 knowledge items, respectively, that were correct and, in the time-pressure conditions, given within the time limit.

#### Numeracy

Numeracy was measured using a combination of the nonadaptive version of the 4-item Berlin Numeracy Test^45^ and the 3-item measure by Schwartz et al.^46^ This combined 7-item measure is recommended for general population online samples.^45^ The numeracy score represents the sum of all correct answers. Cronbach’s α was 0.69.

#### Graph literacy

Graph literacy was measured using the Short Graph Literacy Scale.^47^ It consists of 4 items, which measure the understanding of graphically presented medical information. The graph literacy score represents the sum of all correct answers. Cronbach’s α was 0.33, which is not unusual for a short test that assesses a variety of graph comprehension skills.^47^

#### Demographics

Solely for the purpose of describing the sample, we asked participants for their age, gender, and highest educational degree. We also asked whether they or a close friend or family member had suffered from MS. For exploratory purposes, we also tested whether experience with MS (9.3% of participants) affected the results. Because there was no effect and we did not preregister any analysis regarding MS experience, we did not include it in our analysis.

### Participants

Participants were recruited via Prolific Academic, an online crowdsourcing service. To be eligible, they had to reside in the United States, speak English as their first language, and have a study approval rate of at least 95%. As compensation, participants received £1.50 as a flat fee and a performance-contingent bonus of, on average, £0.80 (*SD* = £0.22). Based on a power analysis using G*Power,^48^ our study required 70 participants per condition to detect a small-to-medium-sized effect (α = 0.05, 1 −β = 0.90, *f* = 0.175). Because of the randomized allocation of medication positions between participants, in the inconsistent conditions the representation formats of the 2 dominant options differed between participants (i.e., both dominant options were represented either numerically or graphically or one was represented numerically and the other graphically). To have the statistical power to test whether the format of the dominant options affected decisions within each inconsistent condition, we aimed to oversample the inconsistent (v. consistent) conditions by a ratio of 3:2, yielding a targeted group size of 105 for the inconsistent conditions. Anticipating a participant exclusion of about 15%, we recruited 704 participants. As preregistered, we excluded 32 participants who failed the attention check, 5 who stated that they had not participated seriously, 1 who repeatedly gave unreasonable answers in the numeracy questionnaire, and 1 who stated in the comments that they noticed that they had misunderstood the task. The final sample consisted of 665 participants and is described in Table 4.

**Table 4:** Sample Description (*N* = 665)^a^

Variable	Mean (*SD*) or %
Gender (%)	
Male	48.6
Female	50.5
Nonbinary/other	0.9
Age (y)	37.5 (13.1)
Education: bachelor’s degree or more (%)	66.2
Experience with multiple sclerosis (%)	9.3
Numeracy (0–7)	3.6 (1.8)
Graph literacy (0–4)	2.5 (1.0)
Response time: decision (s)	50.3 (51.7)
Response time: gist knowledge (s)	21.6 (20.0)
Response time: verbatim knowledge (s)	24.9 (16.2)
Decision accuracy (proportion of correct decisions)	0.76
Gist knowledge	0.74 (0.36)
Verbatim knowledge	0.74 (0.31)

a Descriptive statistics on response times include conditions without time pressure only.

### Data Analysis

To test our Hs and examine our RQs, we conducted a series of linear and logistic regressions. We followed the preregistered analyses for preregistered Hs and RQs and conducted analogous analyses for the Hs and RQs that were not preregistered. We deviated from the preregistration in 2 points: First, in models with knowledge as a dependent variable, we tested whether effects on knowledge differed between verbatim and gist knowledge by including a within-participants knowledge-type variable in the models. Second, when running our preregistered models, which included all possible interactions, unexpected multicollinearity issues arose. To avoid these issues, we included only main effects and 2-way interactions in our models, as our Hs and RQs referred only to those effects. For the analysis of knowledge, we also included knowledge type as a variable and its interactions with the other main effects and 2-way interactions. Results were similar when comparing the preregistered and the revised models. When we examined response times and knowledge, we conducted linear regressions with log-transformed response times (as response time distributions tend to be skewed) and the knowledge scores as outcome variable. For response times, we used data from the no-time-pressure conditions only and analyzed response times for all answers (i.e., both correct and incorrect) in the decision and knowledge questions separately. When we examined decisions, we conducted logistic regressions with correct decision as the outcome variable. As predictors, each regression model included 1 or 2 dummy variables for format, a dummy variable for time pressure (0 = no time pressure, 1 = time pressure), numeracy (mean-centered), graph literacy (mean-centered), all of their 2-way interactions, and, for knowledge models, their interactions with knowledge type (effect coded: −0.5 = verbatim, +0.5 = gist). When comparing icon arrays and numbers (RQ1, RQ2, and RQ3), the format dummy variable was coded as 0 = numerical, 1 = graphical. When we compared inconsistent to consistent representation (H1, H2, H3, and H4), there were 2 dummy variables (dummy 1: 0 = inconsistent, 1 = numerical; dummy 2: 0 = inconsistent, 1 = graphical), with the inconsistent condition being the reference condition. We used 2 dummy variables because we were most interested in the comparison between the inconsistent with the numerical and graphical conditions and because collapsing the numerical and graphical conditions into 1 consistent condition might hide differences between the numerical and graphical conditions. When testing the benefit of the intervention (H5), the predictors were intervention (dummy coded as 0 = no intervention, 1 = intervention), numeracy, graph literacy, all of their 2-way interactions, and their interaction with knowledge type. Here, we analyzed the data of the inconsistent-no-time-pressure condition and intervention condition only. To test whether decisions were biased (H6), we conducted a logistic regression with choice for a graphically presented option as the outcome variable and correct decision (dummy coded as 0 = incorrect decision, 1 = correct decision), numeracy, graph literacy, and all of their 2-way interactions as predictors. By controlling for correct decision, we could identify how the format affected choices beyond whether the choice was correct or not.

We tested the effects of numeracy and graph literacy (RQ4 to RQ7) throughout the models described above. Unless stated otherwise, results were similar when we excluded numeracy and graph literacy as predictors or when we added subjective numeracy and subjective graph literacy as covariates.

## Results

The data and analysis script are openly available at https://osf.io/b5aqk/. Descriptive results, full regressions results, and further results on the separation of incorrect responses into inaccurate and too-slow responses as well as choice proportions for each individual medication can be found in the Supplementary Material. Of the 2 dominant options, participants chose the option with more frequent benefits and side effects more often than the one with less frequent benefits and side effects. For each regression coefficient, we also report a measure of effect size: odds ratios (ORs) for logistic regressions, and $\mu_{p}^{2}$ for linear regressions. According to Cohen,^49^ $\mu_{p}^{2}$ ≥ 0.01, 0.06, and 0.14 as well as OR ≥ 1.44, 2.48, and 4.27 are considered small, medium, and large effect sizes, respectively.

### Time Pressure (H1)

Decision accuracy (*b* = −1.21 [95% confidence interval = −1.79 to −0.66], *P* < 0.001, OR = 0.30) and knowledge (*b* = −0.33 [−0.38 to −0.28], *P* < 0.001, $\mu_{p}^{2}$ = 0.212) were lower in the time-pressure conditions than in the no-time-pressure conditions. This supports our hypothesis that understanding is worse when there is time pressure than when there is no time pressure (H1).

### Numerical versus Graphical Representations

#### Response times (RQ2)

**Figure 4:**
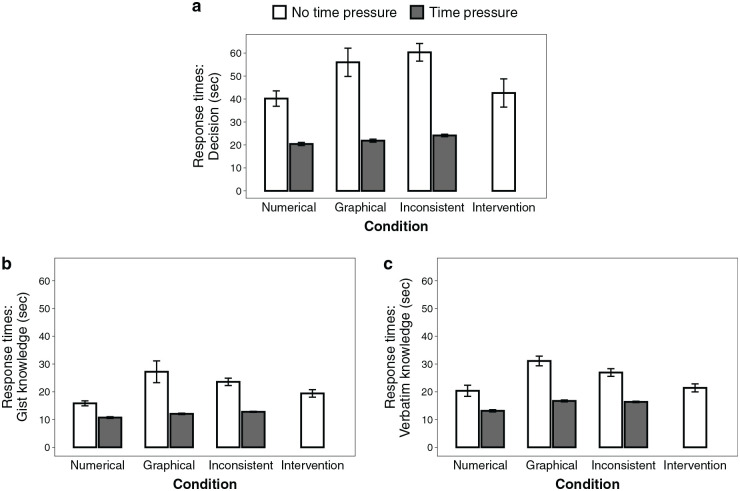
Response times for (a) decisions, (b) gist knowledge, and (c) verbatim knowledge questions. Shown here are the observed, nontransformed means of response times per question (i.e., without controlling for numeracy or graph literacy). Error bars represent 1 standard error of the mean. In the time-pressure conditions, response time was limited (decision: 30 s; knowledge: 15 s for 4 items, 25 s for the other 4 items). Response times in the intervention condition refer to the time taken to answer questions, excluding the time required for the intervention itself.

Response times are illustrated in Figure 4. When information was presented graphically, response times were longer when making decisions (*b* = 0.30 [0.09–0.52], *P* = 0.005, $\mu_{p}^{2}$ = 0.038) and answering knowledge questions (*b* = 0.45 [0.36–0.55], *P* < 0.001, $\mu_{p}^{2}$ = 0.240) than when information was represented numerically (RQ2). Neither numeracy nor graph literacy were related to decision or knowledge response times.

#### Decision accuracy (RQ1, RQ3)

**Figure 5:**
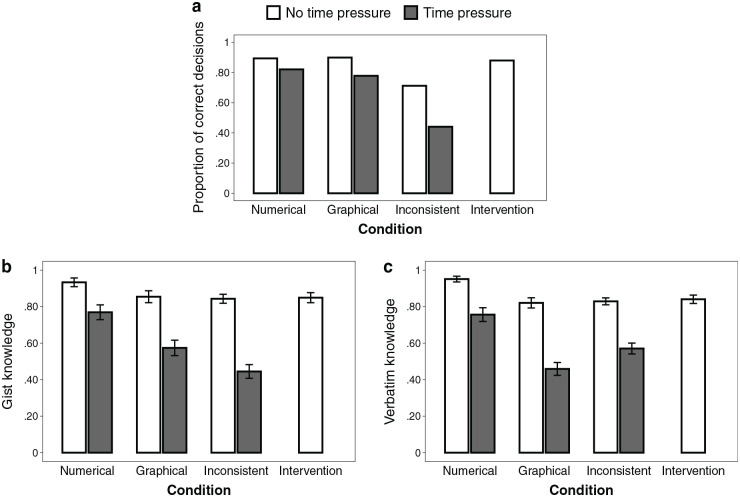
Observed (a) decision accuracy, (b) gist knowledge, and (c) verbatim knowledge across conditions. Error bars represent 1 standard error of the mean.

Decision accuracy is illustrated in Figure 5. Decision accuracy did not differ between formats (RQ1; *b* = −0.13 [−1.47 to 1.14], *P* = 0.84, OR = 0.88). Time pressure did not decrease decision accuracy more strongly when information was represented graphically compared with numerically (RQ3; interaction: *b* = −0.61 [−2.13 to 0.92], *P* = 0.43, OR = 0.54). Numeracy was positively related to decision accuracy (*b* = 0.63 [0.18–1.14], *P* = 0.009, OR = 1.88) but less so in the graphical condition (interaction: *b* = −0.64 [−1.14 to −0.17], *P* = 0.009, OR = 0.53). There was a significant negative interaction between numeracy and graph literacy (*b* = −0.24 [−0.44 to −0.04], *P* = 0.016, OR = 0.79), indicating that numeracy had a greater impact for participants with lower (v. higher) graph literacy. There were no other significant main effects or interactions of numeracy or graph literacy.

#### Knowledge (RQ1, RQ3)

**Figure 6:**
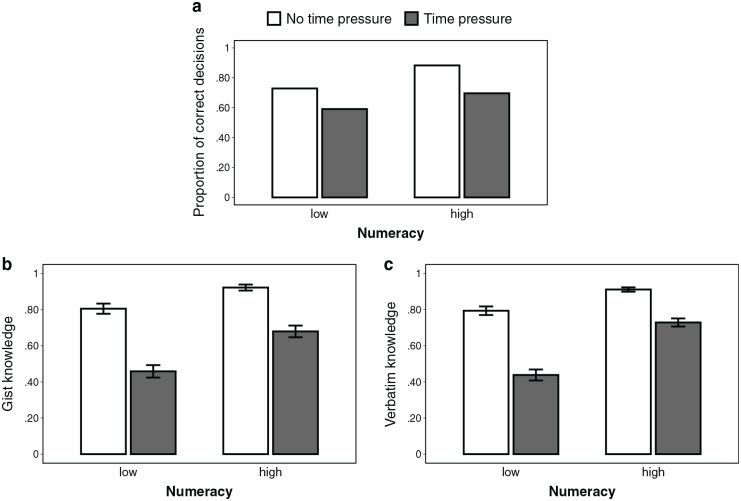
Effect of numeracy on (a) decision accuracy, (b) gist knowledge, and (c) verbatim knowledge, depending on the presence of time pressure, aggregated across format conditions (excluding the intervention condition). For illustrative purposes, we performed a median split for numeracy (0–3 = low, 4–7 = high). However, we used the continuous variable throughout all analyses. Error bars represent 1 standard error of the mean.

Knowledge scores are illustrated in Figure 5. Knowledge was lower when information was presented graphically compared with numerically (RQ1; *b* = −0.09 [−0.15 to −0.03], *P* = 0.006, $\mu_{p}^{2}$ = 0.098), and time pressure decreased knowledge more strongly when information was presented graphically compared with numerically (RQ3; interaction: *b* = −0.15 [−0.24 to −0.06], *P* = 0.001, $\mu_{p}^{2}$ = 0.021). Higher numeracy (*b* = 0.03 [0.01–0.06], *P* = 0.012, $\mu_{p}^{2}$ = 0.113), but not graph literacy (*b* = 0.01 [−0.03 to 0.06], *P* = 0.60, $\mu_{p}^{2}$ = 0.014), tended to increase knowledge. Higher numeracy also attenuated the effect of time pressure (interaction: *b* = 0.04 [0.01–0.07], *P* = 0.003, $\mu_{p}^{2}$ = 0.016; for an illustration of this interaction, see Figure 6). Finally, there was a negative interaction of numeracy and graph literacy (*b* = −0.03 [−0.04 to −0.01], *P* < 0.001, $\mu_{p}^{2}$ = 0.028), indicating that numeracy had a greater impact for participants with lower (v. higher) graph literacy.

### Consistent versus Inconsistent Representation

#### Response times (H3, RQ5)

Response times are shown in Figure 4. In the inconsistent condition, participants took longer to make a decision compared with the numerical condition (*b* = −0.47 [−0.66 to −0.27], *P* < 0.001, $\mu_{p}^{2}$ = 0.052) but not compared with the graphical condition (*b* = −0.16 [−0.35 to 0.03], *P* = 0.103, $\mu_{p}^{2}$ = 0.009). Participants in the inconsistent conditions took longer than participants in the numerical condition to answer knowledge questions (*b* = −0.35 [−0.44 to −0.26], *P* < 0.001, $\mu_{p}^{2}$ = 0.146) but were faster than participants in the graphical condition (*b* = 0.11 [0.02–0.19], *P* = 0.007, $\mu_{p}^{2}$ = 0.015). This provides partial support for our hypothesis that response times are longer with inconsistently represented information than with consistently represented information (H3). Regarding the association of numeracy and graph literacy with response times (RQ5), there were no effects of numeracy or graph literacy on response times in decisions, but participants with higher numeracy responded more quickly to knowledge questions compared with participants with lower numeracy (*b* = −0.07 [−0.11 to −0.04], *P* < 0.001, $\mu_{p}^{2}$ = 0.056), and participants with higher graph literacy tended to take longer to respond compared wth participants with lower graph literacy (*b* = 0.06 [0.00–0.12], *P* = 0.036, $\mu_{p}^{2}$ = 0.004).

#### Decision accuracy (H2, H4, RQ4, RQ6)

Decision accuracy is illustrated in Figure 5. As expected, decisions were less accurate when options were presented inconsistently than when all information was numerical (*b* = 1.64 [0.65–2.83], *P* = 0.003, OR = 5.17) or graphical (*b* = 1.39 [0.56–2.35], *P* = 0.002, OR = 4.03). This supports our hypothesis that understanding in terms of decision accuracy is worse when options are represented inconsistently than when they are represented consistently (H2). Time pressure did not compromise accuracy more strongly in inconsistent than in purely numerical (*b* = 0.77 [−0.52 to 2.03], *P* = 0.23, OR = 2.16) or graphical representations (*b* = 0.17 [−0.95 to 1.24], *P* = 0.75, OR = 1.19). This is not in line with our hypothesis that time pressure decreases understanding in terms of decision accuracy more strongly when information is represented inconsistently (v. consistently; H4). Graph literacy (*b* = 0.41 [0.01–0.83], *P* = 0.048, OR = 1.18), but not numeracy (*b* = 0.17 [−0.06 to 0.41], *P* = 0.156, OR = 1.51), tended to be positively associated with decision accuracy (RQ4). However, numeracy tended to be more strongly associated with accuracy in the numerical condition than in the inconsistent condition (interaction: *b* = 0.45 [0.04–0.90], *P* = 0.038; OR = 1.57). No other interactions with numeracy or graph literacy were significant, indicating that time pressure did not decrease understanding in terms of decision accuracy less strongly for people with higher (v. lower) numeracy or graph literacy (RQ6).

#### Knowledge (H2, H4, RQ4, RQ6)

Knowledge scores are illustrated in Figure 5. Knowledge was lower when information was represented inconsistently than when it was represented numerically (*b* = 0.10 [0.04–0.16], *P* = 0.001, $\mu_{p}^{2}$ = 0.077) but not when it was represented graphically (*b* = 0.01 [−0.05 to 0.07], *P* = 0.72, $\mu_{p}^{2}$ < 0.001). This provides partial support for our hypothesis that understanding in terms of knowledge is worse when options are represented inconsistently (v. consistently; H2). Time pressure decreased gist knowledge more strongly than verbatim knowledge (*b* = −0.14 [−0.24 to −0.04], *P* = 0.006, $\mu_{p}^{2}$ = 0.001). The superiority of numerical over inconsistent representation was even stronger when there was time pressure compared with when there was none (interaction: *b* = 0.17 [0.09–0.25], *P* < 0.001, $\mu_{p}^{2}$ = 0.018). This provides partial support for our hypothesis that time pressure decreases understanding in terms of knowledge more strongly when information is represented inconsistently (v. consistently; H4). Furthermore, this effect tended to be more pronounced for gist knowledge than for verbatim knowledge (3-way interaction: *b* = 0.16 [0.00–0.33], *P* = 0.047, $\mu_{p}^{2}$ = 0.001). There was also a significant format × time × knowledge type 3-way interaction, indicating that for gist knowledge, the effect of time pressure was weaker with graphical representation than with inconsistent representation (*b* = 0.21 [0.05–0.37], *P* = 0.009, $\mu_{p}^{2}$ = 0.007). Both numeracy (*b* = 0.03 [0.01–0.04], *P* = 0.009, $\mu_{p}^{2}$ = 0.114) and graph literacy (*b* = 0.05 [0.02–0.09], *P* = 0.001, $\mu_{p}^{2}$ = 0.016) were positively related to knowledge (RQ4), and the impact of time pressure was lower for people with higher numeracy than for people with lower numeracy (RQ6; interaction: *b* = 0.04 [0.02–0.07], *P* < 0.001, $\mu_{p}^{2}$ = 0.017). Figure 6 illustrates how higher numeracy attenuated the detrimental effect of time pressure for knowledge but not for decisions. There was also a significant negative interaction of numeracy and graph literacy (*b* = −0.02 [−0.03 to −0.01], *P* < 0.001, $\mu_{p}^{2}$ = 0.013), indicating that numeracy had a greater impact for participants with lower (v. higher) graph literacy.

### Intervention

#### Translation accuracy

Overall, participants in the intervention condition on average correctly translated 90.7% (*SD* = 24.6) of the graphically represented information into numbers (77.6% of participants translated all information correctly). In an exploratory analysis, numeracy (*r* = 0.27, *P* = 0.004), but not graph literacy (*r* = 0.17, *P* = 0.066), was found to be positively correlated with translation accuracy.

#### Decision accuracy (H5, RQ7)

Decisions were more accurate in the intervention condition than in the no-intervention conditions (*b* = 1.78 [0.82–2.97], *P* = 0.001, OR = 5.90). This supports the hypothesis that understanding in terms of decision accuracy is better with the intervention than without intervention (H5). Numeracy tended to moderate the effect of intervention (interaction: *b* = 0.62 [0.04–1.27], *P* = 0.046, OR = 1.85), with the intervention leading to a stronger improvement in understanding for people with higher (v. lower) numeracy, although we expected a less strong improvement (RQ7). Further analysis showed that decision accuracy in the intervention condition did not differ from that in the numerical (*b* = 0.55 [−1.12 to 2.73], *P* = 0.55, OR = 1.73) and graphical conditions (*b* = −0.28 [−1.62 to 0.97], *P* = .66, OR = 0.76).

#### Knowledge (H5, RQ7)

There was no difference in knowledge between the intervention and no-intervention conditions (*b* = 0.02 [−0.03 to 0.06], *P* = 0.47, $\mu_{p}^{2}$ < 0.001). This does not support the hypothesis that understanding in terms of knowledge is better with the intervention than without (H5). The effect of intervention was stronger for people with higher (v. lower) numeracy (*b* = 0.03 [0.00–0.06], *P* = 0.025, $\mu_{p}^{2}$ = 0.003), again the opposite of what we expected (RQ7). Knowledge in the intervention condition was lower than in the numerical condition (*b* = 0.08 [0.03–0.13], *P* = 0.001, $\mu_{p}^{2}$ = 0.036) but did not differ from knowledge in the graphical condition (*b* = −0.01 [−0.05 to 0.04], *P* = 0.81, $\mu_{p}^{2}$ < 0.001).

### Choice Bias toward Graphics (H6)

Independent of decision accuracy, graphically represented options were chosen more often than numerically represented ones, indicated by an intercept that was significantly different from 0 (intercept = 0.62 [0.10–1.16], *P* = 0.022, OR = 1.85), which translates into a 64.9% probability of choosing the graphically represented option when controlling for the other predictors. This supports our hypothesis that decisions are biased toward the graphically represented options (H6).

## Discussion

Graphical risk formats have been shown to lead to better understanding of medical information compared with numerical formats.^5–7^ Our research studied how people process information depending on whether it is represented graphically or numerically. Specifically, we focused on how much cognitive effort is required to process different representation formats, how people process medical information when it is represented inconsistently, and how numeracy and graph literacy are related to processing efficiency. In our experiment, participants were provided with information about the benefits and side effects of hypothetical medications and were asked to choose a preferred medication and to answer questions about them. Relative frequencies were either presented as numbers, as icon arrays, or inconsistently (i.e., numbers for some medications and icon arrays for others). We also manipulated whether participants were under time pressure and measured their numeracy and graph literacy.

In line with previous findings,^15,16^ icon arrays led to longer response times than numbers. This could be attributed to people engaging more with graphically represented information or to people having to invest more cognitive resources to understand it—or both. Therefore, neither these studies nor our conditions without time pressure could disentangle whether people *take* more time or *need* more time to process graphically represented information (or both). However, the manipulation of time pressure revealed that increased cognitive effort was at least partly responsible for longer response times: if longer response times were driven solely by increased engagement but information could be processed similarly efficiently, then time pressure should affect understanding in both formats similarly. However, time pressure affected the processing of graphical information more strongly than that of numerical information. These results also held when we compared the time-pressure conditions only, which strengthens our conclusions. Future research should use alternative measures of cognitive load (e.g., letting participants complete a second task simultaneously) to supplement our findings.

Furthermore, we found that when information was represented inconsistently, decisions took longer, were substantially worse, and required more cognitive effort than when all options were represented in the same format. This suggests that with inconsistently represented information, graphs and numbers are processed in different ways and that processing them requires an additional step to integrate information. The results of our intervention condition also showed that people do not seem to deal with inconsistent representations by explicitly translating one representation into another before comparing them, although they are generally capable of doing so. Perhaps, people try to avoid the translation step and instead directly compare the frequencies using an additional internal representation (e.g., categorizing frequencies and then comparing categories), but more research is needed to learn more about the processing of inconsistently represented information. Moreover, when medical information was presented inconsistently, decisions were biased toward the graphically represented options. Thus, our study shows that the representation of information not only affects people’s comprehension of information but can change their treatment preferences as well. Finally, higher numeracy, but not higher graph literacy, was associated with processing information more efficiently.

In contrast to prior studies, we found no benefit of graphical over numerical formats in terms of comprehension. One reason for this deviation could be the relatively large number of icon arrays. Most previous research on icon arrays has only presented a single icon array^7,29^; however, studies that, like ours, presented more than one did not find a benefit of icon arrays compared to numbers.^22,43,50,51^ Future research should systematically study how comprehension of graphically represented information depends on the number of graphics.

The IPDAS guidelines for risk communication recommend transparent graphical formats because of their superiority over numbers in terms of understanding and preferences.^7^ However, our results in conjunction with previous findings suggest that the choice between using numbers or graphs to communicate medical information may be less straightforward than previously assumed. First, when choosing how to present information, there may be a tradeoff to be made between comprehension and cognitive effort: while graphical representations have often been shown to lead to better comprehension, our results suggest that this may come at the cost of higher cognitive effort. Second, graphical representations seem to be more beneficial than numbers for people with higher graph literacy, whereas people with lower graph literacy benefit more from numbers.^40^ Third, previous research has shown that graphical formats are considered more attractive and helpful than numbers.^17,40,42,43,52,53^ However, evidence is mixed on whether format preferences align with comprehension.^11,21,22,53–55^ Finally, the IPDAS guidelines also acknowledge that graphical formats are superior to numbers in terms of gist knowledge, but numbers might be more effective for verbatim knowledge. As a consequence, the goal of the communication—whether it targets gist or verbatim knowledge—may be another important factor to consider when communicating medical information.

One reason why graphical formats are more beneficial for gist knowledge and numbers are more beneficial for verbatim knowledge could be the correspondence between the representation format and the answering format: graphical formats facilitate ordinal comparisons (i.e., gist knowledge), but an additional translation step is necessary to give precise, numerical answers (i.e., verbatim knowledge). Although in our study, participants provided answers in different answering formats depending on whether gist or verbatim knowledge was being tested, this extra translation step could not fully explain why the graphical format required more cognitive effort when answering knowledge questions. If graphical representations required more cognitive effort solely due to differences in the (in)congruencies between the representation and the answering format, answering verbatim knowledge questions using icon arrays should have required more cognitive effort than gist knowledge. This, however, was not the case.

The primary goal of our study was to examine the basic cognitive mechanisms underlying the processing of numerically and graphically represented information. Because it was designed to investigate cognitive mechanisms, the scenario in our study may differ from a real-life decision-making situation. For instance, we decided to present 6 different medications with benefits and side effects, but many patients have fewer treatment options. Nevertheless, for many medical conditions, including MS, more than 6 treatments are available.^56^ Furthermore, we provided an overall relative frequency of side effects for each treatment in our study, although for many medical treatments, different kinds of side effects occur with different frequencies. However, a study that presented multiple side effects (and fewer medications) found similar results to ours: there was no benefit of icon arrays over numbers.^50^ Moreover, we implemented the inconsistent conditions and the intervention to study how people deal with information when the representation format of some options is different from that of other options. Although typically information about all medical treatment options may be represented in the same way, there are situations conceivable in which the representation is less consistent, especially if patients gather information from different sources (e.g., online, physician, government agencies).

Although our study reveals important insights into differences in the processing of numerically and graphically represented information, it can provide only limited inferences about how meaningful these differences are in clinical practice. More research is needed to understand how the way that statistical information is presented affects the decision-making process in real-life settings.

## Limitations

Our study was conducted with a sample from the general population and used hypothetical medical data. Actual patients may process medical information differently than our participants: for instance, patients are likely to be more invested in the decision and therefore to spend more time and cognitive resources. Our study was not designed to determine whether this investment affects information processing, and further research is needed to examine whether our results hold in a patient sample. Because affected patients—due to physical and emotional factors—need even more time to process information, it is possible that time pressure would affect their understanding even stronger than that of the general population and thus the reduction of cognitive effort would be especially imperative for affected patients. On the other hand, is it possible that information processing does not substantially differ between patient and nonpatient samples, as one study found that MS patients have similar levels of numeracy compared with the general population,^57^ and in our study, experience with MS did not affect the results. Second, although the time limits used in this study were carefully selected based on the results of our pilot study, it is possible that different time limits could have led to other results and conclusions. Third, decision accuracy and knowledge scores were relatively high in the conditions without time pressure, and thus, our results may have been subject to ceiling effects, which reduce the ability to detect differences. However, a considerably more difficult task may have been unfeasible under time pressure.

## Conclusion

Studying the cognitive mechanisms underlying the effects of information representation on comprehension is not only important for understanding how different formats affect people’s comprehension of medical information but is also crucial for designing decision aids that aim to both improve people’s medical decisions and facilitate the cognitive processes leading to them. Our findings suggest that numerical and graphical formats may have different strengths and weaknesses that need to be carefully considered when designing decision aids.

## Supplemental Material

sj-docx-1-mdm-10.1177_0272989X231202505 – Supplemental material for How Do People Process Different Representations of Statistical Information? Insights into Cognitive Effort, Representational Inconsistencies, and Individual Differences Click here for additional data file. Supplemental material, sj-docx-1-mdm-10.1177_0272989X231202505 for How Do People Process Different Representations of Statistical Information? Insights into Cognitive Effort, Representational Inconsistencies, and Individual Differences by Kevin E. Tiede and Wolfgang Gaissmaier in Medical Decision Making
