# Supplementary material for: How Do People Process Different Representations of Statistical Information? Insights into Cognitive Effort, Representational Inconsistencies, and Individual Differences
Source: Med Decis Making. 2023 Oct 16;43(7-8):803–20. doi: 10.1177/0272989X231202505 (PMC10625726; doi:10.1177/0272989X231202505)

**Supplemental Materials to the Article**

**How do people process different representations of statistical information? Insights into cognitive effort, representational inconsistencies, and individual differences**

**S1 Screenshots of MS Scenario p2**

**S2 Representation Formats in Different Conditions and Screenshots of the
Intervention and Time-Pressure Conditions p3**

**S3 Description of the Pilot Study p6**

**S4 Descriptive Results and Demographics for Each Condition p8**

**S5 Full Regression Results p9**

**S6 Decision Results for Individual Medication Options p16**

**S7 Responses in the Time-Pressure Conditions Separated by Response Type
(Correct vs. Incorrect vs. Too-Slow) p17**

**S1 Screenshots of MS Scenario**

Figure S1. Screenshots of the description of the Multiple Sclerosis scenario.


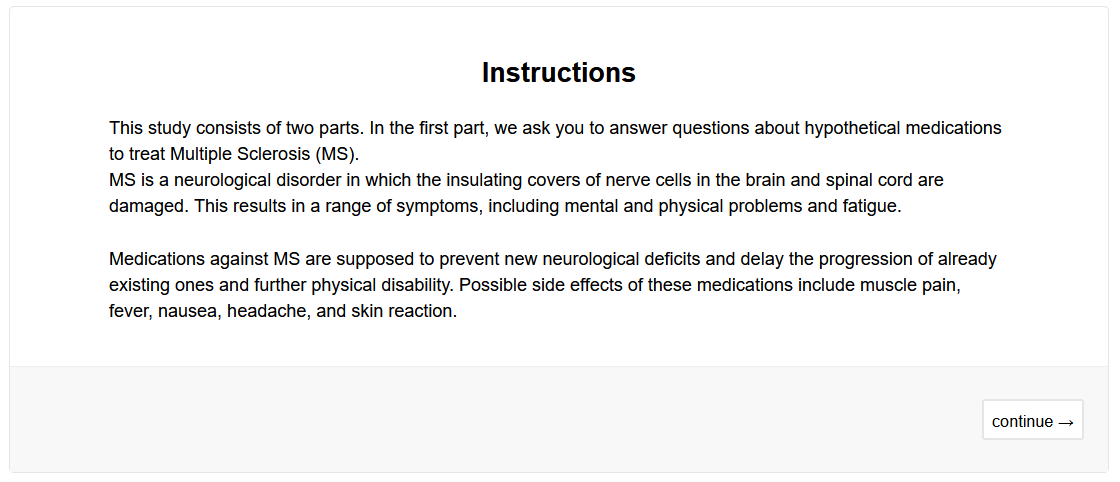


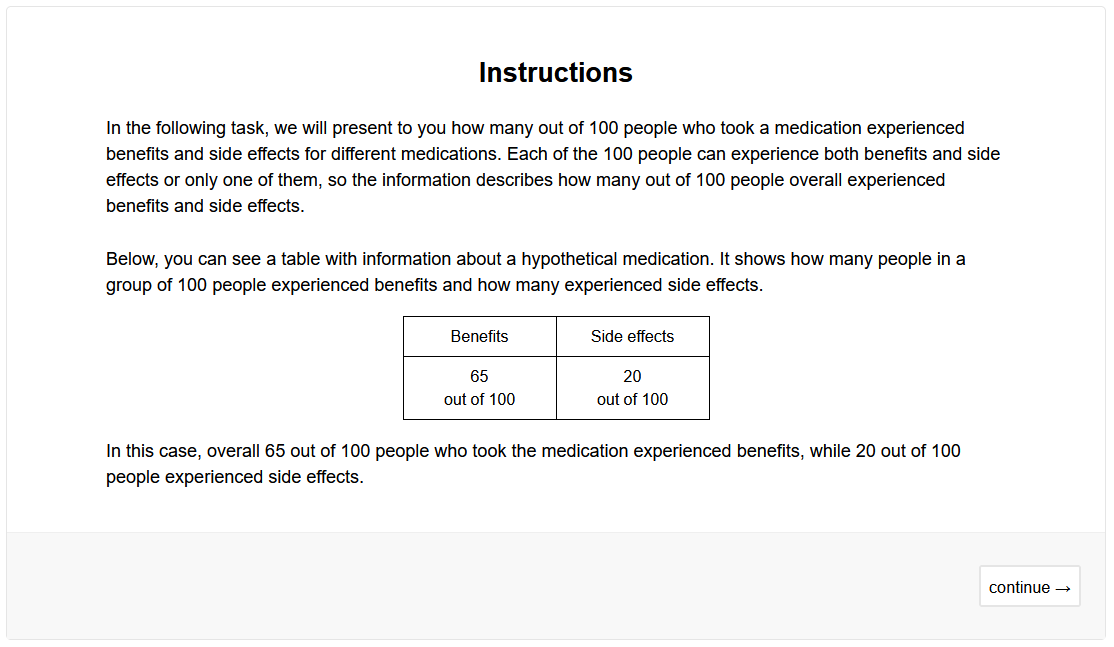


**S2 Representation Formats in Different Conditions and Screenshots of the Intervention and Time-Pressure Conditions**

Figure S2. Medical information as presented in the numerical conditions.

**
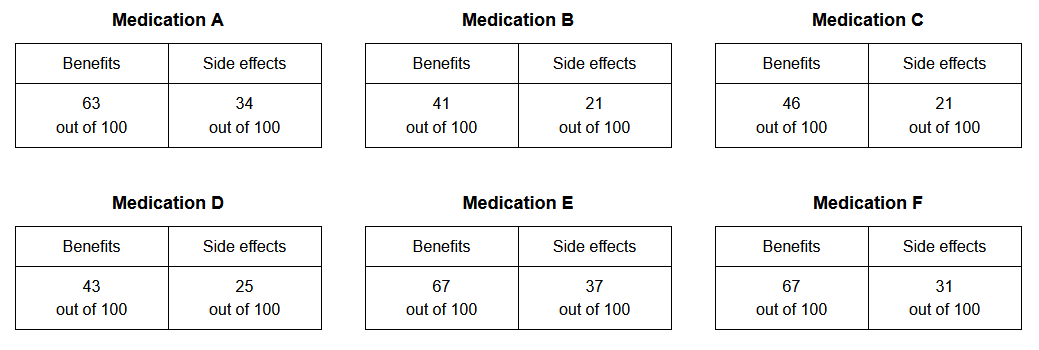
**

Figure S3. Medical information as presented in the graphical conditions.

**
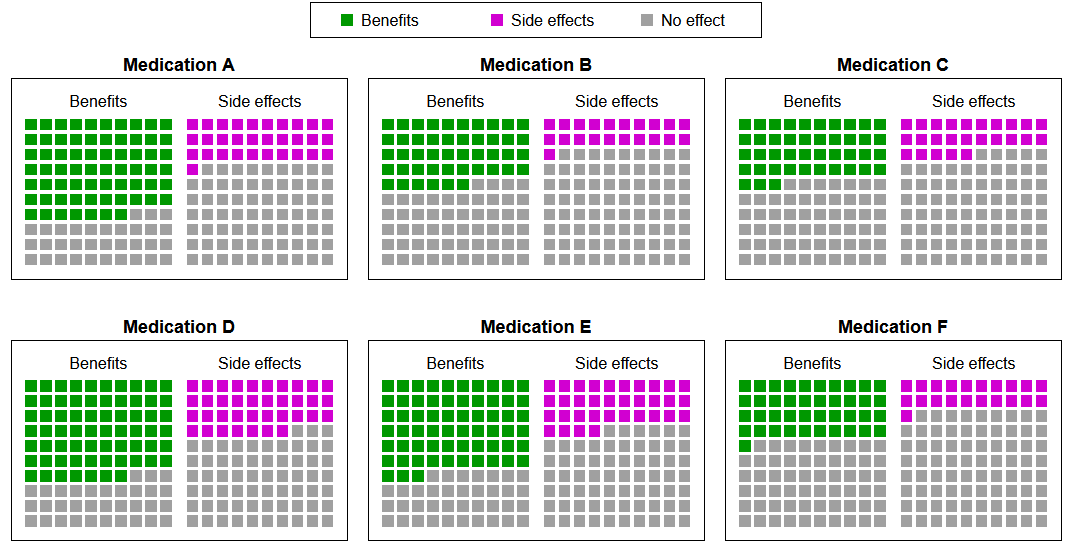
**

Figure S4. Screenshot of the presented information in the intervention condition after inserting the numerical information. The numbers in italics represent the translated numbers as inserted by the participant.


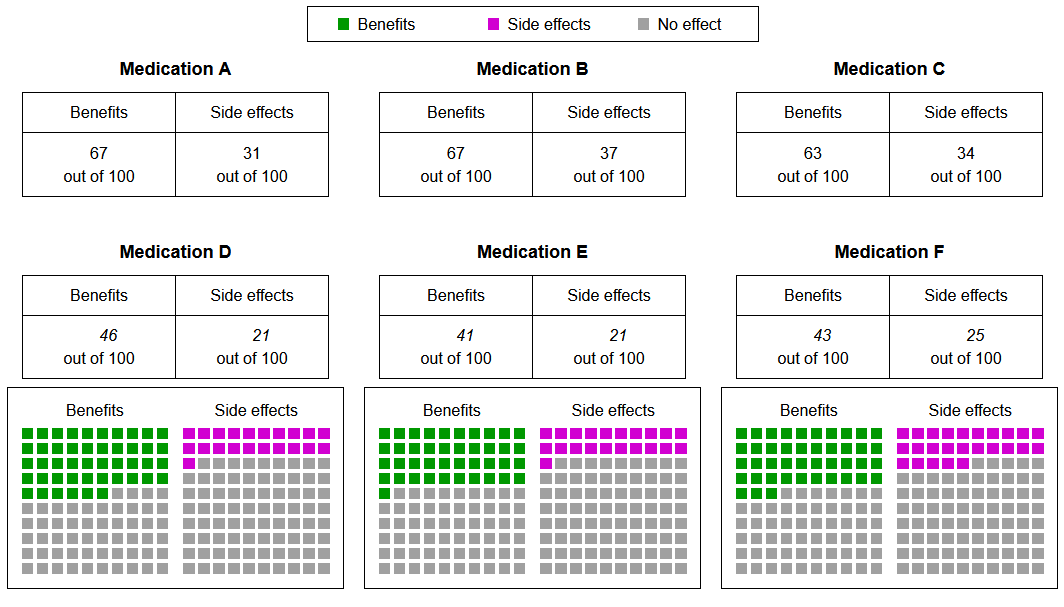


Figure S5. Screenshot of the announcement on how much time participants have to answer the following question in the time-pressure conditions.

**
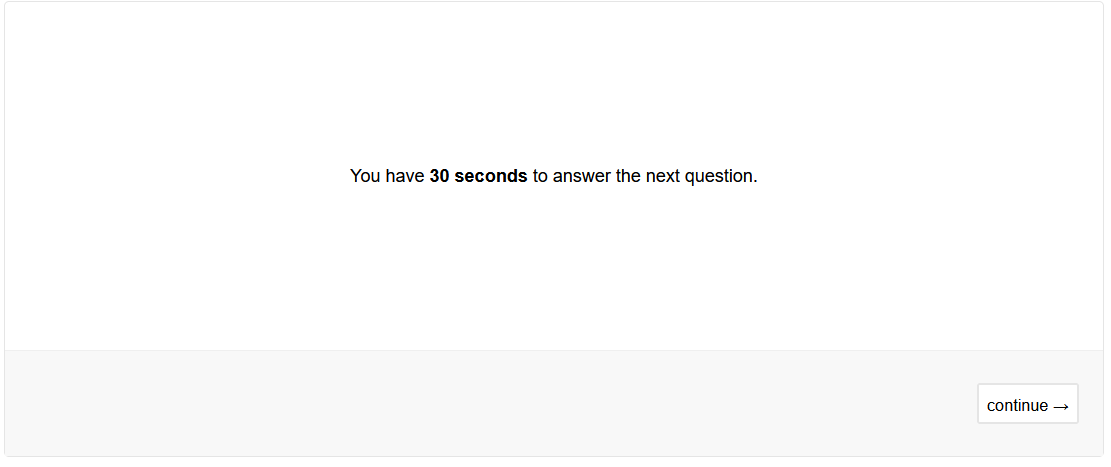
**

Figure S6. Screenshot of a question screen in a condition with time pressure. Note the display of the remaining time in the lower-right corner. When five or fewer seconds were left, the font color of the time presentation turned magenta.

**
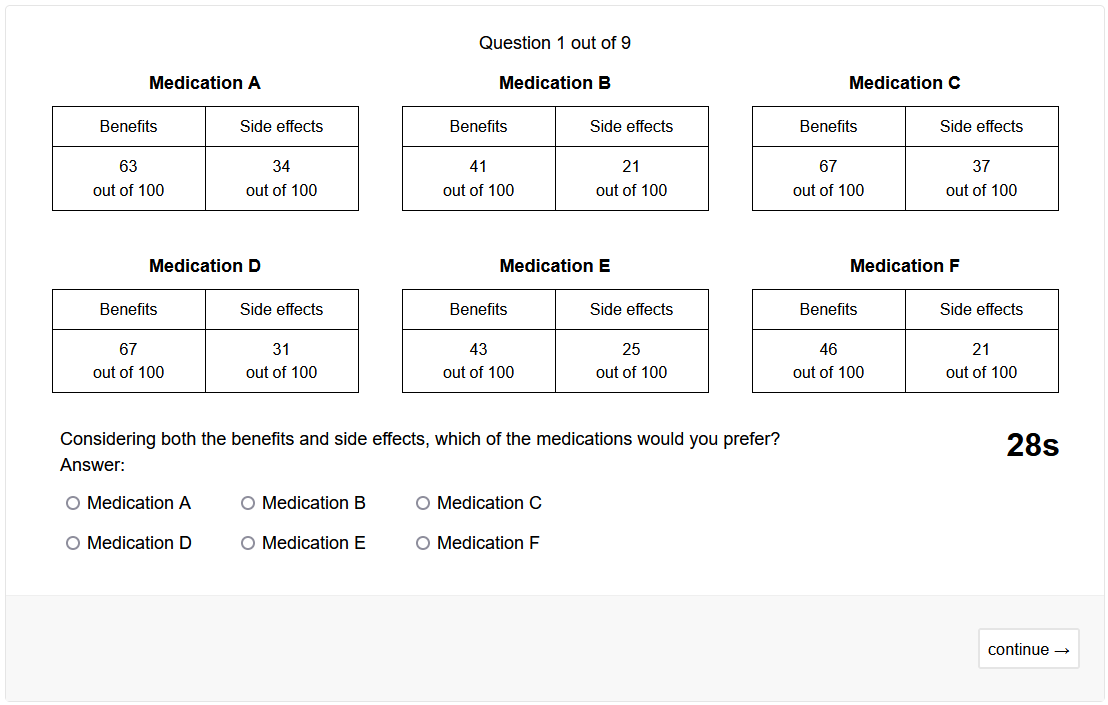
**

**S3 Description of the Pilot Study**

Prior to the main study, we conducted a pilot study to determine appropriate time limits for the time-pressure conditions. We aimed at time limits which urged participants to respond fast but still allowed them to answer the questions in time. The pilot study was similar to the main study with the exception that we only implemented the numerical, graphical, and inconsistent conditions without time pressure. Also, we did not assess numeracy or graph literacy. Sixty participants (34 female, 24 male, 2 non-binary gender, *M*_age_ = 33.0, *SD*_age_ = 12.1, 65.0% college degree or more; *n* = 20 per condition) which were recruited via Prolific Academic took part in the pilot study.

Distributions of response times are presented in Figure S7. For the decision, the median response time was 34.42 seconds (*M* = 41.47, *SD* = 26.39). Participants were faster at answering the knowledge questions, but response times differed considerably between the types of knowledge questions. For gist knowledge questions, the median response time was 14.40 seconds (*M* = 19.21, *SD* = 19.75). For the reading-off items of the verbatim knowledge questions, the median response time was 13.02 seconds (*M* = 15.33, *SD* = 7.62). For the computing-differences items of the verbatim knowledge questions, the median response time was 21.77 seconds (*M* = 24.81, *SD* = 11.81).

For the main study, we aimed at time limits in which about half of the participants would be expected to answer the questions without a time limit. We prioritized rounded numbers with sensible values over hard median cut-offs (i.e., 15 seconds over 13.02 seconds). Therefore, we chose time limits around the median and the mean. Note that we based that time limits on the medians across all participants and conditions and thus all conditions in the main study had the same time limits. We did this to ensure a fair comparison of information processing efficiency between conditions in the main study.

Figure S7. Distribution of response times for (a) decisions, (b) gist knowledge items, (c) verbatim knowledge (reading-off) items, and (d) verbatim knowledge (computing-differences) items. The dashed grey line indicates the time limit chosen for the main study (i.e., 30, 15, 15, and 25 seconds, respectively).

**
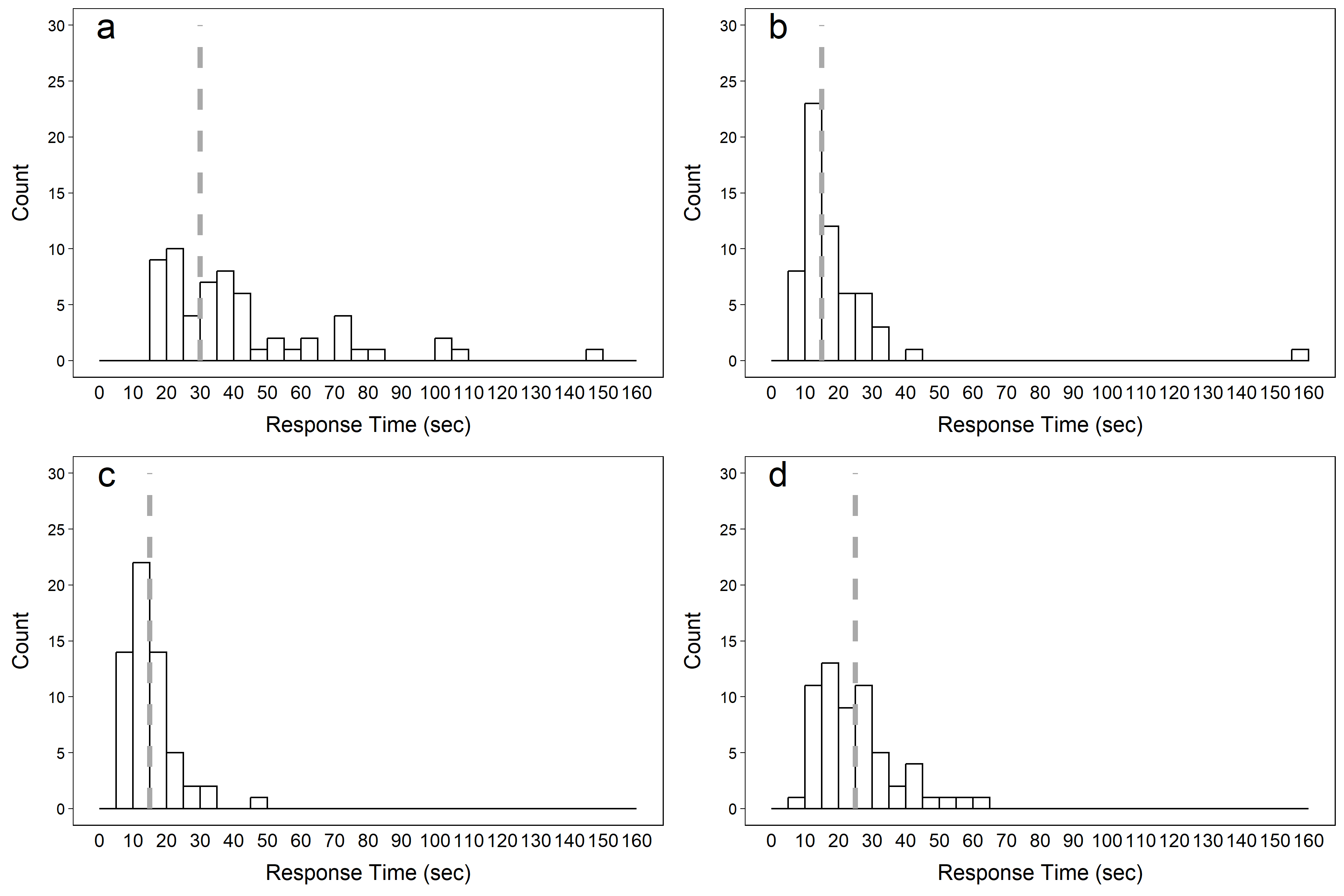
**

**S4 Descriptive Results and Demographics for Each Condition**

Table S1. Descriptive results for each condition. Presented are means (SD) or proportions.

| Variable | Num +  no TP | Graph +  no TP | Incon +  no TP | Num +  TP | Graph + TP | Incon +  TP | Inter-vention |
| --- | --- | --- | --- | --- | --- | --- | --- |
| Group size (*n*) | 75 | 79 | 118 | 78 | 81 | 118 | 116 |
| Gender (%) |  |  |  |  |  |  |  |
| male | 62.7% | 54.4% | 52.5% | 41.0% | 38.3% | 45.8% | 46.6% |
| female | 37.3% | 44.3% | 45.8% | 59.0% | 60.5% | 53.4% | 52.6% |
| nonbinary/  other | 0.0% | 1.3% | 1.7% | 0.0% | 1.2% | 0.8% | 0.9% |
| Age (years) | 35.1 (10.9) | 38.4 (13.8) | 37.7 (14.0) | 38.5 (12.1) | 36.5 (13.0) | 38.7 (13.8) | 36.7 (13.2) |
| Bachelor’s degree or more | 69.3% | 55.7% | 63.6% | 70.5% | 66.7% | 73.7% | 62.9% |
| MS experience | 9.3% | 5.1% | 8.5% | 11.5% | 7.4% | 11.0% | 11.2% |
| Numeracy (0-7) | 3.8 (1.8) | 3.5 (1.9) | 3.7 (1.7) | 3.6 (2.0) | 3.4 (1.8) | 3.8 (1.8) | 3.5 (1.6) |
| Graph literacy (0-4) | 2.8 (0.9) | 2.5 (1.0) | 2.4 (1.0) | 2.4 (1.1) | 2.3 (1.1) | 2.5 (1.0) | 2.6 (0.8) |
| RT: Decision (s) | 40.2 (29.0) | 56.0 (54.7) | 60.3 (41.6) | 20.4 (6.0) | 21.9 (5.9) | 24.1 (5.8) | 42.6 (66.2) |
| RT: Gist knowledge (s) | 15.8 (7.8) | 27.2 (34.9) | 23.6 (14.4) | 10.7 (2.7) | 12.1 (2.3) | 12.8 (2.0) | 19.4 (14.6) |
| RT: Verbatim knowledge (s) | 20.4 (17.4) | 31.1 (15.5) | 26.9 (15.0) | 13.1 (3.6) | 16.7 (3.1) | 16.4 (2.7) | 21.4 (15.6) |
| Decision accuracy | .89 | .90 | .71 | .82 | .78 | .44 | .88 |
| Gist  knowledge | .93 (.21) | .85 (.29) | .84 (.27) | .77 (.36) | .57 (.38) | .44 (.41) | .85 (.30) |
| Verbatim knowledge | .95 (.14) | .82 (.25) | .83 (.21) | .76 (.33) | .46 (.32) | .57 (.32) | .84 (.25) |

Note. RT = Response Times, MS = Multiple Sclerosis, Num = numerical condition, Graph = graphical condition, Incon = inconsistent condition, TP = time pressure present.

**S5 Full Regression Results**

In the following, we present the full regression results of the analyses presented in the main text.

Table S2. Full Results of the Comparison between Numeric vs. Graphical: Response Times (Decisions and Knowledge)

|  | RT: Decision | | |  | RT: Knowledge | | |
| --- | --- | --- | --- | --- | --- | --- | --- |
| Predictor | *b* | *SE* | *p* |  | *b* | *SE* | *p* |
| Intercept | 10.32 | 0.08 | <.001 |  | 9.59 | 0.04 | <.001 |
| NumvsGraph: Num. (0) vs. Graph. (1) | 0.30 | 0.11 | .005 |  | 0.45 | 0.05 | <.001 |
| Numeracy | 0.06 | 0.05 | .230 |  | -0.03 | 0.02 | .164 |
| Graph Literacy | 0.17 | 0.10 | .085 |  | 0.03 | 0.04 | .496 |
| NumvsGraph × Numeracy | -0.07 | 0.07 | .309 |  | -0.05 | 0.03 | .140 |
| NumvsGraph × GraphLit | -0.06 | 0.13 | .613 |  | -0.06 | 0.06 | .348 |
| Numeracy × GraphLit | 0.03 | 0.03 | .312 |  | -0.01 | 0.01 | .336 |
| Type: Verbatim (-0.5) vs. Gist (+0.5) |  |  |  |  | -0.12 | 0.07 | .102 |
| NumvsGraph × Type |  |  |  |  | -0.10 | 0.10 | .308 |
| Numeracy × Type |  |  |  |  | 0.00 | 0.04 | .970 |
| Graph Literacy × Type |  |  |  |  | 0.06 | 0.09 | .484 |
| NumvsGraph × Numeracy × Type |  |  |  |  | 0.03 | 0.06 | .650 |
| NumvsGraph × GraphLit × Type |  |  |  |  | -0.12 | 0.12 | .321 |
| Numeracy × GraphLit × Type |  |  |  |  | 0.01 | 0.03 | .665 |

*Note.* RT = Response Times, Num. = Numerical, Graph. = Graphical, GraphLit = Graph Literacy, Type = Knowledge type.

Table S3. Full Results of the Comparison between Numeric vs. Graphical: Decision Accuracy and Knowledge

|  | Decision | | |  | Knowledge | | |
| --- | --- | --- | --- | --- | --- | --- | --- |
| Predictor | *b* | *SE* | *p* |  | *b* | *SE* | *p* |
| Intercept | 2.74 | 0.52 | <.001 |  | 0.95 | 0.02 | <.001 |
| NumvsGraph: Num. (0) vs. Graph. (1) | -0.13 | 0.65 | .845 |  | -0.09 | 0.03 | .006 |
| Time Pressure: no (0) vs. yes (1) | -0.46 | 0.62 | .454 |  | -0.16 | 0.03 | <.001 |
| Numeracy | 0.63 | 0.24 | .009 |  | 0.03 | 0.01 | .012 |
| Graph Literacy | 0.49 | 0.46 | .286 |  | 0.01 | 0.02 | .595 |
| NumvsGraph × TimePressure | -0.61 | 0.77 | .426 |  | -0.15 | 0.04 | .001 |
| NumvsGraph × Numeracy | -0.64 | 0.25 | .009 |  | -0.01 | 0.01 | .330 |
| TimePressure × Numeracy | -0.07 | 0.24 | .767 |  | 0.04 | 0.01 | .003 |
| NumvsGraph × GraphLit | -0.02 | 0.41 | .956 |  | 0.04 | 0.02 | .084 |
| TimePressure × GraphLit | -0.29 | 0.43 | .492 |  | -0.02 | 0.03 | .529 |
| Numeracy × GraphLit | -0.24 | 0.10 | .016 |  | -0.03 | 0.01 | <.001 |
| Type: Verbatim (-0.5) vs. Gist (+0.5) |  |  |  |  | -0.02 | 0.05 | .653 |
| NumvsGraph × Type |  |  |  |  | 0.05 | 0.06 | .425 |
| TimePressure × Type |  |  |  |  | 0.02 | 0.06 | .705 |
| Numeracy × Type |  |  |  |  | 0.01 | 0.02 | .731 |
| Graph Literacy × Type |  |  |  |  | -0.01 | 0.05 | .803 |
| NumvsGraph × TimePressure × Type |  |  |  |  | 0.04 | 0.09 | .624 |
| NumvsGraph × Numeracy × Type |  |  |  |  | 0.01 | 0.03 | .724 |
| TimePressure × Numeracy × Type |  |  |  |  | -0.00 | 0.03 | .890 |
| NumvsGraph × GraphLit × Type |  |  |  |  | -0.04 | 0.05 | .448 |
| TimePressure × GraphLit × Type |  |  |  |  | -0.04 | 0.05 | .400 |
| Numeracy × GraphLit × Type |  |  |  |  | 0.01 | 0.01 | .606 |

*Note.* Num. = Numerical, Graph. = Graphical, GraphLit = Graph Literacy, Type = Knowledge type.

Table S4. Full Results of the Comparison between Inconsistent vs. Consistent: Response Times (Decisions and Knowledge)

|  | RT: Decision | | |  | RT: Knowledge | | |
| --- | --- | --- | --- | --- | --- | --- | --- |
| Predictor | *b* | *SE* | *p* |  | *b* | *SE* | *p* |
| Intercept | 10.80 | 0.06 | <.001 |  | 9.94 | 0.03 | <.001 |
| InconsVsNum: Incons. (0) vs. Num. (1) | -0.47 | 0.10 | <.001 |  | -0.35 | 0.05 | <.001 |
| InconsVsGraph: Incons. vs. Graph. (1) | -0.16 | 0.10 | .103 |  | 0.11 | 0.04 | .016 |
| Numeracy | -0.02 | 0.04 | .692 |  | -0.07 | 0.02 | <.001 |
| Graph Literacy | 0.11 | 0.07 | .094 |  | 0.06 | 0.03 | .036 |
| InconsVsNum × Numeracy | 0.08 | 0.06 | .219 |  | 0.04 | 0.03 | .143 |
| InconsVsGraph × Numeracy | 0.01 | 0.06 | .907 |  | -0.00 | 0.03 | .894 |
| InconsVsNum × GraphLit | 0.06 | 0.12 | .625 |  | -0.03 | 0.05 | .537 |
| InconsVsGraph × GraphLit | -0.02 | 0.11 | .876 |  | -0.09 | 0.05 | .070 |
| Numeracy × GraphLit | 0.01 | 0.02 | .707 |  | -0.02 | 0.01 | .121 |
| Type: Verbatim (-0.5) vs. Gist (+0.5) |  |  |  |  | -0.05 | 0.06 | .345 |
| InconsVsNum × Type |  |  |  |  | -0.06 | 0.09 | .509 |
| InconsVsGraph × Type |  |  |  |  | -0.16 | 0.09 | .072 |
| Numeracy × Type |  |  |  |  | 0.02 | 0.03 | .645 |
| Graph Literacy × Type |  |  |  |  | 0.07 | 0.06 | .265 |
| InconsVsNum × Numeracy × Type |  |  |  |  | -0.01 | 0.06 | .821 |
| InconsVsGraph × Numeracy × Type |  |  |  |  | 0.01 | 0.06 | .796 |
| InconsVsNum × GraphLit × Type |  |  |  |  | -0.01 | 0.11 | .963 |
| InconsVsGraph × GraphLit × Type |  |  |  |  | -0.13 | 0.10 | .203 |
| Numeracy × GraphLit × Type |  |  |  |  | 0.00 | 0.02 | .925 |

*Note.* RT = Response Times, Incons. = Inconsistent, Num. = Numerical, Graph. = Graphical, GraphLit = Graph Literacy, Type = Knowledge type.

Table S5. Full Results of the Comparison between Inconsistent vs. Consistent: Decision Accuracy and Knowledge

|  | Decision | | |  | Knowledge | | |
| --- | --- | --- | --- | --- | --- | --- | --- |
| Predictor | *b* | *SE* | *p* |  | *b* | *SE* | *p* |
| Intercept | 1.02 | 0.22 | <.001 |  | 0.85 | 0.02 | <.001 |
| InconsVsNum: Incons. (0) vs. Num. (1) | 1.64 | 0.55 | .003 |  | 0.10 | 0.03 | .001 |
| InconsVsGraph: Incons. vs. Graph. (1) | 1.39 | 0.45 | .002 |  | 0.01 | 0.03 | .723 |
| Time Pressure: no (0) vs. yes (1) | -1.21 | 0.29 | <.001 |  | -0.33 | 0.03 | <.001 |
| Numeracy | 0.17 | 0.12 | .156 |  | 0.03 | 0.01 | .009 |
| Graph Literacy | 0.41 | 0.21 | .048 |  | 0.05 | 0.02 | .001 |
| InconsVsNum × TimePressure | 0.77 | 0.65 | .232 |  | 0.17 | 0.04 | <.001 |
| InconsVsGraph × TimePressure | 0.17 | 0.55 | .753 |  | 0.03 | 0.04 | .493 |
| InconsVsNum × Numeracy | 0.45 | 0.22 | .038 |  | 0.00 | 0.01 | .893 |
| InconsVsGraph × Numeracy | -0.18 | 0.17 | .274 |  | -0.01 | 0.01 | .448 |
| TimePressure × Numeracy | -0.03 | 0.14 | .831 |  | 0.04 | 0.01 | <.001 |
| InconsVsNum × GraphLit | 0.33 | 0.35 | .351 |  | -0.04 | 0.02 | .106 |
| InconsVsGraph × GraphLit | 0.14 | 0.28 | .621 |  | 0.00 | 0.02 | .881 |
| TimePressure × GraphLit | -0.34 | 0.25 | .166 |  | -0.02 | 0.02 | .397 |
| Numeracy × GraphLit | -0.08 | 0.07 | .211 |  | -0.02 | 0.00 | <.001 |
| Type: Verbatim (-0.5) vs. Gist (+0.5) |  |  |  |  | 0.01 | 0.04 | .793 |
| InconsVsNum × Type |  |  |  |  | -0.03 | 0.06 | .569 |
| InconsVsGraph × Type |  |  |  |  | 0.02 | 0.06 | .793 |
| TimePressure × Type |  |  |  |  | -0.14 | 0.05 | .006 |
| Numeracy × Type |  |  |  |  | -0.01 | 0.02 | .505 |
| Graph Literacy × Type |  |  |  |  | 0.02 | 0.03 | .637 |
| InconsVsNum × TimePressure × Type |  |  |  |  | 0.16 | 0.08 | .047 |
| InconsVsGraph × TimePressure × Type |  |  |  |  | 0.21 | 0.08 | .009 |
| InconsVsNum × Numeracy × Type |  |  |  |  | 0.02 | 0.03 | .431 |
| InconsVsGraph × Numeracy × Type |  |  |  |  | 0.03 | 0.03 | .209 |
| TimePressure × Numeracy × Type |  |  |  |  | -0.00 | 0.02 | .830 |
| InconsVsNum × GraphLit × Type |  |  |  |  | -0.03 | 0.05 | .533 |
| InconsVsGraph × GraphLit × Type |  |  |  |  | -0.07 | 0.04 | .121 |
| TimePressure × GraphLit × Type |  |  |  |  | -0.03 | 0.04 | .356 |
| Numeracy × GraphLit × Type |  |  |  |  | 0.01 | 0.01 | .260 |

*Note.* Incons. = Inconsistent, Num. = Numerical, Graph. = Graphical, GraphLit = Graph Literacy, Type = Knowledge type.

Table S6. Full Results on the Effect of Intervention

|  | Decision | | |  | Knowledge | | |
| --- | --- | --- | --- | --- | --- | --- | --- |
| Predictor | *b* | *SE* | *p* |  | *b* | *SE* | *p* |
| Intercept | 0.96 | 0.22 | <.001 |  | 0.85 | 0.02 | <.001 |
| Intervention: No (0) vs. Yes (1) | 1.78 | 0.54 | .001 |  | 0.02 | 0.02 | .486 |
| Numeracy | 0.17 | 0.14 | .196 |  | 0.02 | 0.01 | .026 |
| Graph Literacy | 0.47 | 0.24 | .050 |  | 0.06 | 0.02 | .001 |
| Intervention × Numeracy | 0.62 | 0.31 | .046 |  | 0.03 | 0.02 | .025 |
| Intervention × GraphLit | 0.03 | 0.51 | .946 |  | -0.06 | 0.03 | .044 |
| Numeracy × GraphLit | 0.06 | 0.13 | .640 |  | -0.02 | 0.01 | .020 |
| Type: Verbatim (-0.5) vs. Gist (+0.5) |  |  |  |  | 0.01 | 0.03 | .677 |
| Intervention × Type |  |  |  |  | -0.01 | 0.05 | .801 |
| Numeracy × Type |  |  |  |  | -0.01 | 0.02 | .508 |
| Graph Literacy × Type |  |  |  |  | 0.01 | 0.03 | .721 |
| Intervention × Numeracy × Type |  |  |  |  | -0.00 | 0.03 | .919 |
| Intervention × GraphLit × Type |  |  |  |  | 0.01 | 0.06 | .825 |
| Numeracy × GraphLit × Type |  |  |  |  | 0.00 | 0.01 | .768 |

*Note.* GraphLit = Graph Literacy. Type = Knowledge type.

Table S7. Full Results of the Choice Bias Analysis

| Predictor | *b* | *SE* | *p* |
| --- | --- | --- | --- |
| Intercept | 0.62 | 0.27 | .022 |
| Decision Accuracy: incorrect (0) vs. correct (1) | -0.29 | 0.32 | .359 |
| Numeracy | 0.09 | 0.16 | .561 |
| Graph Literacy | 0.08 | 0.26 | .744 |
| Accuracy × Numeracy | -0.19 | 0.20 | .345 |
| Accuracy × GraphLit | -0.31 | 0.34 | .363 |
| Numeracy × GraphLit | 0.05 | 0.10 | .591 |

*Note.* GraphLit = Graph Literacy.

**S6 Decision Results for Individual Medication Options**

In the main text, we report results on decisions based on their accuracy because decision accuracy was one of the primary outcome variables. To provide more detailed results on which particular mediations participants chose, in Table S8 we present the choice proportions for each individual medication option, both overall and separated by condition. Medications 1 and 4 in Table S8 were the dominant (i.e., Pareto optimal) medications. As a reminder, the order in which the medications were presented (i.e., the allocation of the medications) was randomized for each participant, so that particularly in the inconsistent conditions, the probability of each medication presented numerically or graphically was the same.

Table S8. Number (and proportion) of participants who chose each medication (see Table 2 in main text).

| Represen-tation | Time pressure | *n* | **Med. 1** | Med. 2 | Med. 3 | **Med. 4** | Med. 5 | Med. 6 |
| --- | --- | --- | --- | --- | --- | --- | --- | --- |
| Numerical | no | 75 | 62  (82.7%) | 4  (5.3%) | 3  (4%) | 5  (6.7%) | 1  (1.3%) | 0  (0%) |
| Numerical | yes | 70 | 59  (84.3%) | 2  (2.9%) | 2  (2.9%) | 5  (7.1%) | 2  (2.9%) | 0  (0%) |
| Graphical | no | 79 | 60  (75.9%) | 2  (2.5%) | 3  (3.8%) | 11  (13.9%) | 2  (2.5%) | 1  (1.3%) |
| Graphical | yes | 69 | 51  (73.9%) | 3  (4.3%) | 1  (1.4%) | 12  (17.4%) | 2  (2.9%) | 0  (0%) |
| Inconsistent | no | 118 | 78  (66.1%) | 15  (12.7%) | 10  (8.5%) | 6  (5.1%) | 5  (4.2%) | 4  (3.4%) |
| Inconsistent | yes | 88 | 42  (47.7%) | 18  (20.5%) | 8  (9.1%) | 10  (11.4%) | 7  (8.0%) | 3  (3.4%) |
| Intervention | no | 116 | 94  (81.0%) | 7  (6%) | 5  (4.3%) | 8  (6.9%) | 0  (0%) | 2  (1.7%) |
| Total |  | 615 | 446  (72.5%) | 51  (8.3%) | 32  (5.2%) | 57  (9.3%) | 19  (3.1%) | 10  (1.6%) |

*Note.* Med. = Medication. Medication numbering refers to numbering used in Table 2 in the main text. Med. 1 and Med. 4 are the superior medications (i.e., Pareto optimal) and thus are printed in boldface. Results only include decisions which were made within the time limit in the time-pressure conditions and thus may deviate from the group sizes reported in the results on decision accuracy.

**S7 Responses in the Time-Pressure Conditions Separated by Response Type (Correct vs. Incorrect vs. Too-Slow)**

As specified in the preregistration, we considered responses as incorrect if either the response was incorrect or, in the time-pressure condition, the response not submitted within the time limit. As an exploratory analysis, we also examined responses separated by whether they were correct, incorrect, and too slow. As illustrated in Figure S8, the proportion of too-slow decisions were greater in the inconsistent conditions than in the purely numerical or graphical condition. With regard to knowledge, the proportion of too-slow responses is greater in the graphical and the inconsistent conditions than in the numerical condition. This pattern is in line with the results presented in the main text which suggest that more cognitive resources are required when working with inconsistent representation (vs. consistent ones) when making decisions and with graphical and inconsistent representations (vs. numerical representations) when answering knowledge questions.

Figure S8. Observed proportions of correct vs. incorrect vs. too-slow responses for (a) decisions, (b) gist knowledge, and (c) verbatim knowledge questions.


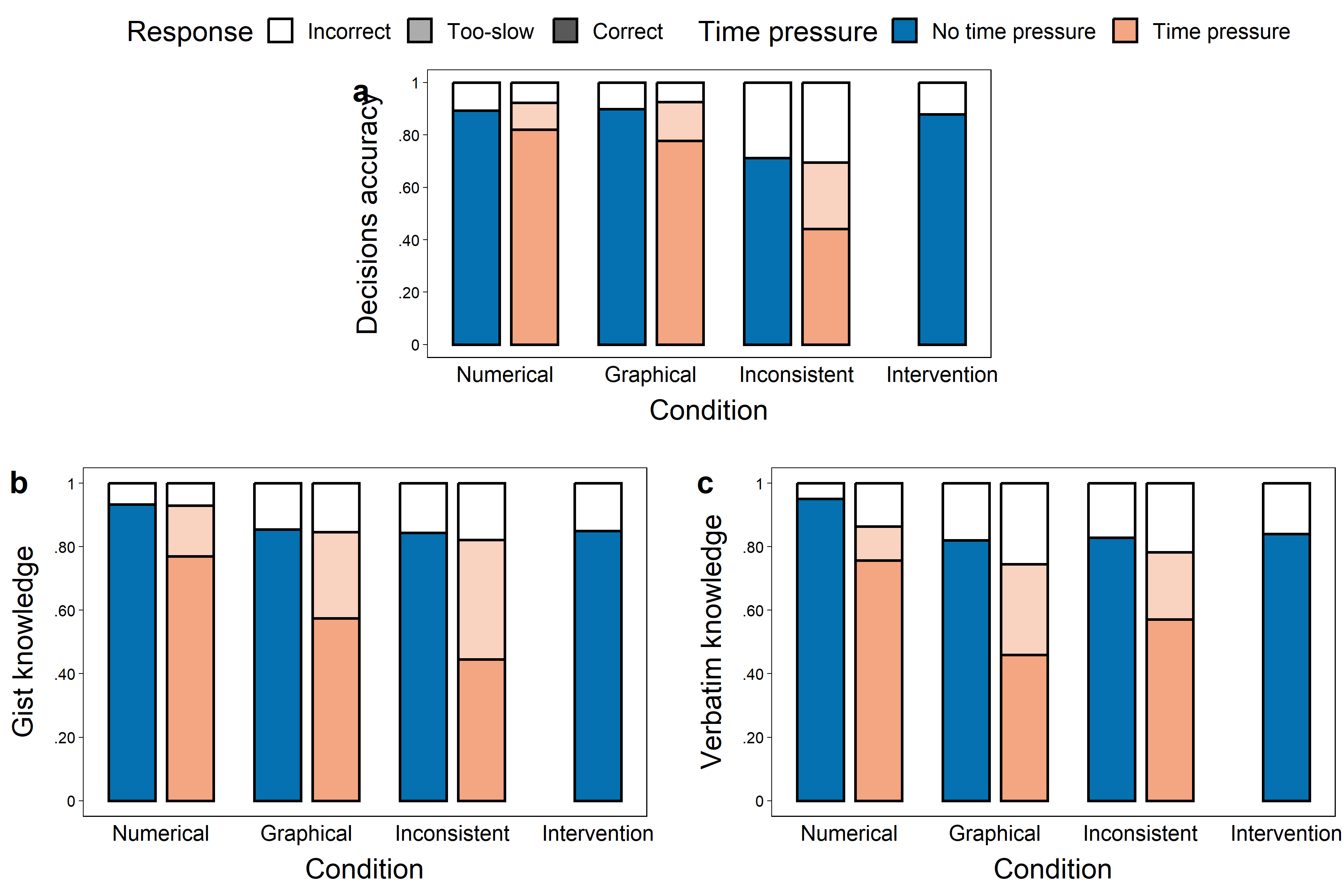

Supplement: sj-docx-1-mdm-10.1177_0272989X231202505 – Supplemental material for How Do People Process Different Representations of Statistical Information? Insights into Cognitive Effort, Representational Inconsistencies, and Individual Differences [file sj-docx-1-mdm-10.1177_0272989X231202505.docx]
